# Poisoning with the per Chloride of Mercury

**Published:** 1852-03

**Authors:** W. W. Goff

**Affiliations:** York, Michigan


### ARTICLE V.

*Poisoning with Per Chloride of Mercury.* By W. W. Goff, M. D., of York, Michigan.

I send you the notes of a case of poisoning with this preparation, which occurred in the practice of Dr. Bowers, of this place.

W. Richards, aged 22, nervous sanguine temperament, good constitution, a farmer; on the 28th of August last, being slightly indisposed, thought to take some salts, and says he took a spoonful dissolved in water, of what he supposed to be Epsom Salts. Soon after swallowing the dose, he felt heat and burning in the fauces and stomach, with sickness and anxiety ; on some examination he discovered that he had taken Corrosive Sublimate. In the consternation of the moment he swallowed three or four ounces of Castor Oil, and more or less Boneset tea, for the purpose of inducing vomiting. This not producing emesis he started for the Drs. office, a mile and a half distant ; at which place I saw him, in consultation, at 3 o'clock P. M., an hour and a half after the poison was swallowed. At this time his face was swelled, somewhat livid ; lips purple and swelled ; eyes suffused and blodshot ; with great difficuliy in articulation. Dr. B. had administered an emetic of Ipecac, the influence of which he was under at the time ; its action was assisted by copious draughts of warm water, and full emesis was soon induced ; the retching continued for some hours after the proper action of the emetic, with ejections of thick tenacious mucus ; probably this came as much from the first passages, as from the stomach. At this time pulse small, almost thready ; great anxiety of countenance, and pain in præcordium ; surface warm ; tongue some enlarged and completely coated with slate colored fur.

As soon as he was free of the emetic, albumen—whites of eggs and flour, was freely administered. The eggs and flour were beat together, and he took as much as he could keep down. Two dozen eggs were given during the first three hours, and two dozen more, that night and the next day. After the first dose the symptoms abated gradually ; less nausea, less retching and anxiety. The bowels moved while he was vomiting, and he had several dejections during the afternoon, very fetid. The albumen was continued, with short intervals during the afternoon and evening ; also milk to drink.

At 6 o'clock some reaction was evident, and the patient was allowed cold water freely; thirst was a constant symptom; but fluids were allowed but sparingly, on account of the tendency to vomit, when the stomach was distended.

The next day profuse salivation was present, swelling of the parotids, &c., also watery diarrhœa, with some admixture of blood, and shreds and patches of mucus membrane, with tenderness of the abdomen. These symptoms were treated with astringents and fomentations, and continued for several days.

The patient soon recovered his usual good health, and is now enjoying a honey moon with a loving bride.

*York, Michigan, Feb.*, 1852.

